# Sodium *N*-chloro-2-methyl­benzene­sulfonamidate sesquihydrate

**DOI:** 10.1107/S1600536809019989

**Published:** 2009-05-29

**Authors:** B. Thimme Gowda, Sabine Foro, Hartmut Fuess

**Affiliations:** a Department of Chemistry, Mangalore University, Mangalagangotri 574 199, Mangalore, India; b Institute of Materials Science, Darmstadt University of Technology, Petersenstrasse 23, D-64287 Darmstadt, Germany

## Abstract

In the title salt, Na^+^·C_7_H_7_ClNO_2_S^−^·1.5H_2_O, one of the water mol­ecules lies on a twofold axis. The sodium ion shows an O_6_ octa­hedral coordination defined by three water O atoms and three sulfonyl O atoms derived from three different anions. The S—N distance of 1.5898 (19) Å is consistent with an S=N double bond. The crystal structure is stabilized by N—H⋯O and O—H⋯Cl hydrogen bonds.

## Related literature

For background to *N*-halo-aryl­sulfonamides, see: Gowda *et al.* (2005 ▶). For related structures, see: Gowda *et al.* (2007*a* ▶,*b* ▶,*c* ▶); George *et al.* (2000 ▶); Olmstead & Power (1986 ▶).
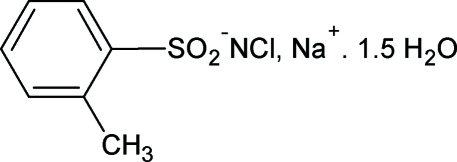


## Experimental

### 

#### Crystal data

Na^+^·C_7_H_7_ClNO_2_S^−^·1.5H_2_O
*M* *_r_* = 509.32
Monoclinic, 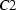

*a* = 11.011 (1) Å
*b* = 6.6434 (6) Å
*c* = 14.447 (1) Å
β = 100.350 (7)°
*V* = 1039.61 (15) Å^3^
*Z* = 2
Mo *K*α radiation
μ = 0.60 mm^−1^
*T* = 299 K
0.45 × 0.32 × 0.08 mm

#### Data collection

Oxford Diffraction Xcalibur diffractometer with a Sapphire CCD detector
Absorption correction: multi-scan (*CrysAlis RED*; Oxford Diffraction, 2007 ▶) *T*_min_ = 0.776, *T*_max_ = 0.954
3268 measured reflections
1657 independent reflections
1613 reflections with *I* > 2σ(*I*)
*R*_int_ = 0.018

#### Refinement

*R*[*F* ^2^ > 2σ(*F* ^2^)] = 0.023
*wR*(*F* ^2^) = 0.061
*S* = 1.02
1657 reflections
142 parameters
1 restraint
H atoms treated by a mixture of independent and constrained refinement
Δρ_max_ = 0.25 e Å^−3^
Δρ_min_ = −0.16 e Å^−3^
Absolute structure: Flack (1983 ▶), 617 Friedel pairs
Flack parameter: 0.02 (6)

### 

Data collection: *CrysAlis CCD* (Oxford Diffraction, 2004 ▶); cell refinement: *CrysAlis RED* (Oxford Diffraction, 2007 ▶); data reduction: *CrysAlis RED*; program(s) used to solve structure: *SHELXS97* (Sheldrick, 2008 ▶); program(s) used to refine structure: *SHELXL97* (Sheldrick, 2008 ▶); molecular graphics: *PLATON* (Spek, 2009 ▶); software used to prepare material for publication: *SHELXL97*.

## Supplementary Material

Crystal structure: contains datablocks I, global. DOI: 10.1107/S1600536809019989/tk2451sup1.cif

Structure factors: contains datablocks I. DOI: 10.1107/S1600536809019989/tk2451Isup2.hkl

Additional supplementary materials:  crystallographic information; 3D view; checkCIF report

## Figures and Tables

**Table 1:** Hydrogen-bond geometry (Å, °)

*D*—H⋯*A*	*D*—H	H⋯*A*	*D*⋯*A*	*D*—H⋯*A*
O3—H31⋯Cl1^i^	0.83 (3)	2.62 (3)	3.4266 (19)	167 (3)
O3—H32⋯N1^ii^	0.77 (3)	2.19 (3)	2.951 (3)	168 (3)
O4—H41⋯N1^iii^	0.80 (3)	2.19 (3)	2.989 (2)	176 (3)

Symmetry codes: (i) 
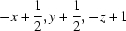
; (ii) 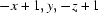; (iii) 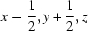.

**Fig. 1.**
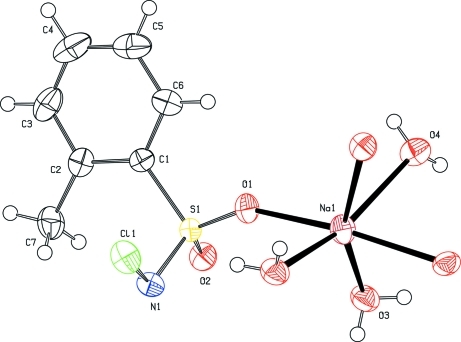
Molecular structure of (I), extended to show the immediate coordination geometry of the sodium cation. Only atoms comprising the asymmetric unit are labelled. Displacement ellipsoids are drawn at the 50% probability level.

**Fig. 2.**
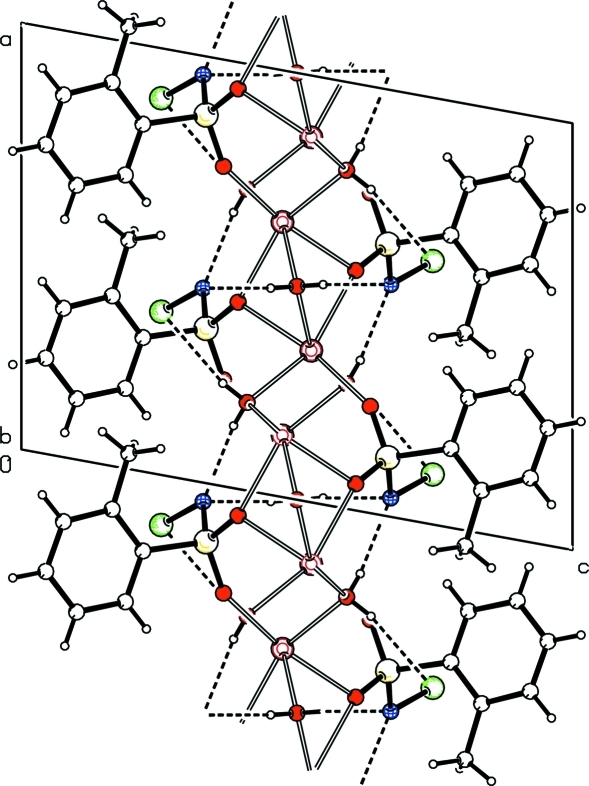
Part of the crystal structure of (I) as viewed down the b axis, showing the O3W—H31···Cl1, O3W—H32···N1 and O4W—H41···N1 hydrogen bonds as dashed lines.

## supplementary crystallographic information

### Comment

The chemistry of *N*-halo-arylsulfonamides is of interest due to their diverse characteristics (Gowda *et al.*, 2005). In the present work, the structure of sodium *N*-chloro-2-methyl- benzenesulfonamidate (I) has been determined to explore substituent effects on the solid-state structures of N-halo arylsulfonamidates (Gowda *et al.*, 2007*a*, *b*, *c*). The structure of (I), Fig. 1, resembles the sodium salts of *N*-chloro-4-chloro-benzenesulfonamidate (Gowda *et al.*, 2007a), *N*-chloro-2-methyl-4-chloro-benzenesulfonamidate (Gowda *et al.*, 2007*b*), *N*-bromo-benzenesulfonamidate (Gowda *et al.*, 2007c), and other sodium *N*-chloro-arylsulfonamidates (George *et al.*, 2000; Olmstead & Power, 1986). The sodium ion shows octahedral coordination defined by three water-O atoms and by three sulfonyl-O atoms derived from three different anions. There is no interaction between the nitrogen and sodium ions in (I). The N1—S1 distance of 1.5898 (19) Å is consistent with a S—N double bond and is in agreement with those observed with related *N*-chloro arylsulfonamides. In this description, the negative charge on the anion is distributed over the oxygen atoms. With the association described above for the Na^+^ ion along with N-H···O and O-H···Cl hydrogen bonding, Table 1, the molecular packing comprises layers stacked along the c axis (Fig. 2).

### Experimental

The purity of the commmercial sample (TCI Chemicals, Tokyo Kasei) was checked and characterized by recording its infrared and NMR spectra and estimating the amount of active chlorine present in it by the iodometric method (Gowda *et al.*, 2005). The single crystals used in X-ray diffraction studies were grown in a water solution of (I) by slow evaporation at room temperature.

### Refinement

The O-bound H atoms were located in difference map and their positional parameters were refined freely [O—H = 0.77 (3)– 0.83 (3) Å]. The other H atoms were positioned in their idealized geometry using a riding model  [C—H = 0.93–0.96 Å]. All H atoms were refined with isotropic displacement parameters set to 1.2 times of the *U*_eq_ of the parent atom.

### Crystal data

**Table:**

Na^+^·C_7_H_7_ClNO_2_S^−^·1.5H_2_O	*F*(000) = 524
*M**_r_* = 509.32	*D*_x_ = 1.627 Mg m^−^^3^
Monoclinic, *C*2	Mo *K*α radiation, λ = 0.71073 Å
Hall symbol: C 2y	Cell parameters from 3049 reflections
*a* = 11.011 (1) Å	θ = 2.6–27.5°
*b* = 6.6434 (6) Å	µ = 0.60 mm^−^^1^
*c* = 14.447 (1) Å	*T* = 299 K
β = 100.350 (7)°	Plate, colourless
*V* = 1039.61 (15) Å^3^	0.45 × 0.32 × 0.08 mm
*Z* = 2	

### Data collection

**Table:**

Oxford Diffraction Xcalibur diffractometer with a Sapphire CCD detector	1657 independent reflections
Radiation source: fine-focus sealed tube	1613 reflections with *I* > 2σ(*I*)
graphite	*R*_int_ = 0.018
Rotation method data acquisition using ω and φ scans	θ_max_ = 25.3°, θ_min_ = 2.9°
Absorption correction: multi-scan (*CrysAlis RED*; Oxford Diffraction, 2007)	*h* = −13→12
*T*_min_ = 0.776, *T*_max_ = 0.954	*k* = −7→8
3268 measured reflections	*l* = −16→17

### Refinement

**Table:**

Refinement on *F*^2^	Secondary atom site location: difference Fourier map
Least-squares matrix: full	Hydrogen site location: inferred from neighbouring sites
*R*[*F*^2^ > 2σ(*F*^2^)] = 0.023	H atoms treated by a mixture of independent and constrained refinement
*wR*(*F*^2^) = 0.061	*w* = 1/[σ^2^(*F*_o_^2^) + (0.041*P*)^2^ + 0.5427*P*] where *P* = (*F*_o_^2^ + 2*F*_c_^2^)/3
*S* = 1.02	(Δ/σ)_max_ = 0.017
1657 reflections	Δρ_max_ = 0.25 e Å^−^^3^
142 parameters	Δρ_min_ = −0.16 e Å^−^^3^
1 restraint	Absolute structure: Flack (1983), 617 Friedel pairs
Primary atom site location: structure-invariant direct methods	Flack parameter: 0.02 (6)

### Special details

**Table:**

Experimental. Absorption correction: CrysAlis RED, Oxford Diffraction Ltd., 2007 Empirical absorption correction using spherical harmonics, implemented in SCALE3 ABSPACK scaling algorithm.
Geometry. All e.s.d.'s (except the e.s.d. in the dihedral angle between two l.s. planes) are estimated using the full covariance matrix. The cell e.s.d.'s are taken into account individually in the estimation of e.s.d.'s in distances, angles and torsion angles; correlations between e.s.d.'s in cell parameters are only used when they are defined by crystal symmetry. An approximate (isotropic) treatment of cell e.s.d.'s is used for estimating e.s.d.'s involving l.s. planes.
Refinement. Refinement of *F*^2^ against ALL reflections. The weighted *R*-factor *wR* and goodness of fit *S* are based on *F*^2^, conventional *R*-factors *R* are based on *F*, with *F* set to zero for negative *F*^2^. The threshold expression of *F*^2^ > σ(*F*^2^) is used only for calculating *R*-factors(gt) *etc*. and is not relevant to the choice of reflections for refinement. *R*-factors based on *F*^2^ are statistically about twice as large as those based on *F*, and *R*- factors based on ALL data will be even larger.

### Fractional atomic coordinates and isotropic or equivalent isotropic displacement parameters (Å^2^)

**Table:**

	*x*	*y*	*z*	*U*_iso_*/*U*_eq_	
Cl1	0.38269 (5)	0.04628 (9)	0.25363 (4)	0.03904 (16)	
S1	0.36260 (4)	0.41295 (8)	0.33507 (3)	0.02316 (13)	
Na1	0.14461 (8)	0.19582 (15)	0.47389 (6)	0.0333 (2)	
O1	0.24978 (13)	0.3583 (3)	0.36677 (10)	0.0367 (4)	
O2	0.43766 (13)	0.5621 (3)	0.39333 (10)	0.0320 (4)	
O3	0.29326 (15)	0.3727 (3)	0.59025 (12)	0.0365 (4)	
H31	0.258 (3)	0.402 (5)	0.6345 (18)	0.044*	
H32	0.354 (3)	0.324 (5)	0.615 (2)	0.044*	
O4	0.0000	0.4675 (4)	0.5000	0.0358 (6)	
H41	−0.015 (3)	0.534 (5)	0.4534 (18)	0.043*	
N1	0.45553 (16)	0.2319 (3)	0.32989 (12)	0.0286 (4)	
C1	0.31412 (18)	0.5172 (3)	0.22046 (13)	0.0254 (4)	
C2	0.3987 (2)	0.5799 (4)	0.16471 (15)	0.0310 (5)	
C3	0.3489 (3)	0.6595 (4)	0.07650 (16)	0.0442 (6)	
H3	0.4026	0.7045	0.0380	0.053*	
C4	0.2246 (3)	0.6740 (5)	0.04438 (17)	0.0533 (7)	
H4	0.1954	0.7255	−0.0153	0.064*	
C5	0.1428 (3)	0.6125 (5)	0.10021 (19)	0.0503 (7)	
H5	0.0582	0.6233	0.0788	0.060*	
C6	0.1876 (2)	0.5343 (4)	0.18857 (16)	0.0367 (5)	
H6	0.1328	0.4930	0.2268	0.044*	
C7	0.5365 (2)	0.5657 (5)	0.19499 (17)	0.0410 (6)	
H7A	0.5600	0.4270	0.2040	0.049*	
H7B	0.5610	0.6378	0.2529	0.049*	
H7C	0.5763	0.6233	0.1473	0.049*	

### Atomic displacement parameters (Å^2^)

**Table:**

	*U*^11^	*U*^22^	*U*^33^	*U*^12^	*U*^13^	*U*^23^
Cl1	0.0411 (3)	0.0278 (3)	0.0496 (3)	−0.0037 (2)	0.0118 (2)	−0.0106 (3)
S1	0.0232 (2)	0.0237 (3)	0.0231 (2)	−0.0006 (2)	0.00549 (16)	−0.0007 (2)
Na1	0.0316 (5)	0.0327 (5)	0.0374 (4)	−0.0053 (4)	0.0114 (4)	−0.0002 (4)
O1	0.0316 (8)	0.0456 (12)	0.0360 (8)	−0.0025 (7)	0.0143 (6)	0.0047 (7)
O2	0.0343 (8)	0.0302 (10)	0.0305 (7)	−0.0028 (7)	0.0034 (6)	−0.0084 (6)
O3	0.0270 (8)	0.0421 (12)	0.0396 (8)	0.0008 (7)	0.0040 (6)	−0.0010 (7)
O4	0.0457 (14)	0.0257 (14)	0.0342 (11)	0.000	0.0025 (10)	0.000
N1	0.0262 (9)	0.0240 (11)	0.0342 (9)	−0.0005 (7)	0.0016 (7)	−0.0019 (7)
C1	0.0296 (10)	0.0206 (12)	0.0250 (9)	−0.0003 (9)	0.0024 (7)	−0.0009 (8)
C2	0.0383 (12)	0.0250 (14)	0.0306 (10)	−0.0045 (9)	0.0091 (8)	−0.0017 (9)
C3	0.0646 (17)	0.0364 (17)	0.0332 (11)	−0.0040 (13)	0.0132 (11)	0.0056 (11)
C4	0.076 (2)	0.0450 (18)	0.0334 (12)	0.0053 (15)	−0.0048 (13)	0.0079 (12)
C5	0.0449 (15)	0.0508 (18)	0.0478 (14)	0.0069 (12)	−0.0115 (11)	−0.0001 (12)
C6	0.0319 (11)	0.0337 (14)	0.0432 (11)	0.0019 (10)	0.0033 (9)	0.0007 (11)
C7	0.0357 (12)	0.0449 (17)	0.0457 (12)	−0.0071 (11)	0.0163 (10)	0.0029 (12)

### Geometric parameters (Å, °)

**Table:**

Cl1—N1	1.7491 (19)	O3—H31	0.83 (3)
S1—O1	1.4454 (15)	O3—H32	0.77 (3)
S1—O2	1.4571 (16)	O4—Na1^iv^	2.480 (2)
S1—N1	1.5898 (19)	O4—H41	0.80 (3)
S1—C1	1.785 (2)	C1—C6	1.391 (3)
S1—Na1^i^	3.3517 (10)	C1—C2	1.399 (3)
Na1—O1	2.3549 (17)	C2—C3	1.397 (3)
Na1—O3	2.4283 (18)	C2—C7	1.505 (3)
Na1—O2^ii^	2.4319 (16)	C3—C4	1.367 (4)
Na1—O4	2.480 (2)	C3—H3	0.9300
Na1—O3^ii^	2.483 (2)	C4—C5	1.375 (4)
Na1—O2^iii^	2.5266 (16)	C4—H4	0.9300
Na1—S1^ii^	3.3517 (10)	C5—C6	1.384 (4)
Na1—Na1^iv^	3.4021 (16)	C5—H5	0.9300
Na1—Na1^i^	4.0447 (10)	C6—H6	0.9300
Na1—Na1^ii^	4.0447 (10)	C7—H7A	0.9600
O2—Na1^i^	2.4319 (16)	C7—H7B	0.9600
O2—Na1^v^	2.5266 (16)	C7—H7C	0.9600
O3—Na1^i^	2.483 (2)		
			
O1—S1—O2	114.72 (9)	O3—Na1—Na1^ii^	88.59 (6)
O1—S1—N1	114.96 (10)	O2^ii^—Na1—Na1^ii^	80.53 (5)
O2—S1—N1	103.80 (9)	O4—Na1—Na1^ii^	158.72 (5)
O1—S1—C1	105.10 (9)	O3^ii^—Na1—Na1^ii^	34.12 (4)
O2—S1—C1	108.43 (10)	O2^iii^—Na1—Na1^ii^	103.70 (5)
N1—S1—C1	109.72 (9)	S1^ii^—Na1—Na1^ii^	58.91 (2)
O1—S1—Na1^i^	74.39 (7)	Na1^iv^—Na1—Na1^ii^	119.16 (2)
N1—S1—Na1^i^	125.01 (7)	Na1^i^—Na1—Na1^ii^	110.42 (4)
C1—S1—Na1^i^	119.92 (8)	S1—O1—Na1	151.10 (10)
O1—Na1—O3	83.29 (6)	S1—O2—Na1^i^	116.81 (8)
O1—Na1—O2^ii^	169.34 (7)	S1—O2—Na1^v^	151.20 (10)
O3—Na1—O2^ii^	86.05 (6)	Na1^i^—O2—Na1^v^	86.63 (5)
O1—Na1—O4	99.86 (6)	Na1—O3—Na1^i^	110.90 (7)
O3—Na1—O4	85.06 (6)	Na1—O3—H31	107 (2)
O2^ii^—Na1—O4	78.79 (5)	Na1^i^—O3—H31	107 (2)
O1—Na1—O3^ii^	87.14 (7)	Na1—O3—H32	123 (2)
O3—Na1—O3^ii^	118.69 (5)	Na1^i^—O3—H32	105 (2)
O2^ii^—Na1—O3^ii^	98.34 (7)	H31—O3—H32	103 (3)
O4—Na1—O3^ii^	156.00 (6)	Na1^iv^—O4—Na1	86.61 (9)
O1—Na1—O2^iii^	111.58 (6)	Na1^iv^—O4—H41	119 (2)
O3—Na1—O2^iii^	158.28 (7)	Na1—O4—H41	108 (2)
O2^ii^—Na1—O2^iii^	78.57 (6)	S1—N1—Cl1	109.69 (10)
O4—Na1—O2^iii^	77.02 (5)	C6—C1—C2	121.0 (2)
O3^ii^—Na1—O2^iii^	79.06 (6)	C6—C1—S1	116.98 (16)
O1—Na1—S1^ii^	152.30 (5)	C2—C1—S1	122.02 (15)
O3—Na1—S1^ii^	79.28 (5)	C3—C2—C1	116.5 (2)
O2^ii^—Na1—S1^ii^	22.83 (4)	C3—C2—C7	119.9 (2)
O4—Na1—S1^ii^	99.90 (4)	C1—C2—C7	123.7 (2)
O3^ii^—Na1—S1^ii^	82.61 (5)	C4—C3—C2	122.7 (2)
O2^iii^—Na1—S1^ii^	91.68 (5)	C4—C3—H3	118.6
O1—Na1—Na1^iv^	137.40 (5)	C2—C3—H3	118.6
O3—Na1—Na1^iv^	112.82 (5)	C3—C4—C5	120.1 (2)
O2^ii^—Na1—Na1^iv^	47.85 (4)	C3—C4—H4	119.9
O4—Na1—Na1^iv^	46.69 (4)	C5—C4—H4	119.9
O3^ii^—Na1—Na1^iv^	114.48 (5)	C4—C5—C6	119.3 (2)
O2^iii^—Na1—Na1^iv^	45.53 (4)	C4—C5—H5	120.3
S1^ii^—Na1—Na1^iv^	69.87 (2)	C6—C5—H5	120.3
O1—Na1—Na1^i^	54.03 (5)	C5—C6—C1	120.4 (2)
O3—Na1—Na1^i^	34.99 (5)	C5—C6—H6	119.8
O2^ii^—Na1—Na1^i^	115.82 (6)	C1—C6—H6	119.8
O4—Na1—Na1^i^	74.73 (4)	C2—C7—H7A	109.5
O3^ii^—Na1—Na1^i^	126.33 (6)	C2—C7—H7B	109.5
O2^iii^—Na1—Na1^i^	144.54 (5)	H7A—C7—H7B	109.5
S1^ii^—Na1—Na1^i^	113.86 (3)	C2—C7—H7C	109.5
Na1^iv^—Na1—Na1^i^	119.16 (2)	H7A—C7—H7C	109.5
O1—Na1—Na1^ii^	99.54 (6)	H7B—C7—H7C	109.5
			
O2—S1—O1—Na1	−71.5 (3)	O2^ii^—Na1—O4—Na1^iv^	−41.12 (4)
N1—S1—O1—Na1	48.8 (2)	O3^ii^—Na1—O4—Na1^iv^	44.21 (14)
C1—S1—O1—Na1	169.5 (2)	O2^iii^—Na1—O4—Na1^iv^	39.58 (4)
Na1^i^—S1—O1—Na1	−73.0 (2)	S1^ii^—Na1—O4—Na1^iv^	−49.86 (2)
O3—Na1—O1—S1	49.7 (2)	Na1^i^—Na1—O4—Na1^iv^	−162.08 (4)
O2^ii^—Na1—O1—S1	51.7 (5)	Na1^ii^—Na1—O4—Na1^iv^	−54.89 (12)
O4—Na1—O1—S1	133.4 (2)	O1—S1—N1—Cl1	58.78 (12)
O3^ii^—Na1—O1—S1	−69.7 (2)	O2—S1—N1—Cl1	−175.11 (9)
O2^iii^—Na1—O1—S1	−146.7 (2)	C1—S1—N1—Cl1	−59.39 (13)
S1^ii^—Na1—O1—S1	−1.5 (3)	Na1^i^—S1—N1—Cl1	146.70 (6)
Na1^iv^—Na1—O1—S1	166.35 (18)	O1—S1—C1—C6	3.1 (2)
Na1^i^—Na1—O1—S1	70.6 (2)	O2—S1—C1—C6	−119.99 (19)
Na1^ii^—Na1—O1—S1	−37.8 (2)	N1—S1—C1—C6	127.3 (2)
O1—S1—O2—Na1^i^	−2.28 (14)	Na1^i^—S1—C1—C6	−77.3 (2)
N1—S1—O2—Na1^i^	−128.55 (10)	O1—S1—C1—C2	−177.07 (18)
C1—S1—O2—Na1^i^	114.82 (10)	O2—S1—C1—C2	59.8 (2)
O1—S1—O2—Na1^v^	139.18 (19)	N1—S1—C1—C2	−52.9 (2)
N1—S1—O2—Na1^v^	12.9 (2)	Na1^i^—S1—C1—C2	102.50 (18)
C1—S1—O2—Na1^v^	−103.7 (2)	C6—C1—C2—C3	0.0 (3)
Na1^i^—S1—O2—Na1^v^	141.5 (3)	S1—C1—C2—C3	−179.79 (19)
O1—Na1—O3—Na1^i^	30.30 (8)	C6—C1—C2—C7	179.9 (2)
O2^ii^—Na1—O3—Na1^i^	−149.33 (8)	S1—C1—C2—C7	0.2 (3)
O4—Na1—O3—Na1^i^	−70.26 (7)	C1—C2—C3—C4	−0.9 (4)
O3^ii^—Na1—O3—Na1^i^	113.32 (11)	C7—C2—C3—C4	179.1 (3)
O2^iii^—Na1—O3—Na1^i^	−104.58 (18)	C2—C3—C4—C5	1.3 (5)
S1^ii^—Na1—O3—Na1^i^	−171.33 (7)	C3—C4—C5—C6	−0.6 (5)
Na1^iv^—Na1—O3—Na1^i^	−108.70 (6)	C4—C5—C6—C1	−0.3 (5)
Na1^ii^—Na1—O3—Na1^i^	130.07 (7)	C2—C1—C6—C5	0.6 (4)
O1—Na1—O4—Na1^iv^	149.64 (6)	S1—C1—C6—C5	−179.6 (2)
O3—Na1—O4—Na1^iv^	−128.06 (5)		

Symmetry codes: (i) −*x*+1/2, *y*+1/2, −*z*+1; (ii) −*x*+1/2, *y*−1/2, −*z*+1; (iii) *x*−1/2, *y*−1/2, *z*; (iv) −*x*, *y*, −*z*+1; (v) *x*+1/2, *y*+1/2, *z*.

### Hydrogen-bond geometry (Å, °)

**Table:**

*D*—H···*A*	*D*—H	H···*A*	*D*···*A*	*D*—H···*A*
O3—H31···Cl1^i^	0.83 (3)	2.62 (3)	3.4266 (19)	167 (3)
O3—H32···N1^vi^	0.77 (3)	2.19 (3)	2.951 (3)	168 (3)
O4—H41···N1^vii^	0.80 (3)	2.19 (3)	2.989 (2)	176 (3)

Symmetry codes: (i) −*x*+1/2, *y*+1/2, −*z*+1; (vi) −*x*+1, *y*, −*z*+1; (vii) *x*−1/2, *y*+1/2, *z*.
